# Adaptive response to l‐serine deficiency is mediated by p38 MAPK activation via 1‐deoxysphinganine in normal fibroblasts

**DOI:** 10.1002/2211-5463.12038

**Published:** 2016-03-03

**Authors:** Tomoko Sayano, Yuki Kawano, Wataru Kusada, Yashiho Arimoto, Kayoko Esaki, Momoko Hamano, Miyako Udono, Yoshinori Katakura, Takuya Ogawa, Hisanori Kato, Yoshio Hirabayashi, Shigeki Furuya

**Affiliations:** ^1^ Laboratory of Functional Genomics and MetabolismDepartment of Innovative Science and Technology for Bio‐industryGraduate School of Bioresource and Bioenvironmental SciencesKyushu UniversityFukuokaJapan; ^2^ Laboratory for Molecular Membrane NeuroscienceRIKEN Brain Science InstituteWakoSaitamaJapan; ^3^ Department of Bioscience and BiotechnologyGraduate School of Bioresource and Bioenvironmental SciencesKyushu UniversityFukuokaJapan; ^4^ Department of Genetic Resources TechnologyGraduate School of Bioresource and Bioenvironmental SciencesKyushu UniversityFukuokaJapan; ^5^ Department of Pharmaceutical SciencesInternational University of Health and WelfareTochigiJapan; ^6^ Corporate Sponsored Research Program ‘Food for Life’, Organization for Interdisciplinary Research ProjectsThe University of TokyoJapan

**Keywords:** cell proliferation, nutritional stress, p21, p38 MAPK, serine deficiency

## Abstract

Reduced availability of l‐serine limits cell proliferation and leads to an adaptation to l‐serine‐deficient environment, the underlying molecular mechanism of which remain largely unexplored. Genetic ablation of 3‐phosphoglycerate dehydrogenase (Phgdh), which catalyzes the first step of *de novo* l‐serine synthesis, led to diminished cell proliferation and the activation of p38 MAPK and stress‐activated protein kinase/Jun amino‐terminal kinase in mouse embryonic fibroblasts under l‐serine depletion. The resultant l‐serine deficiency induced cyclin‐dependent kinase inhibitor 1a (Cdkn1a; p21) expression, which was mediated by p38 MAPK. Survival of the *Phgdh*‐deficient mouse embryonic fibroblasts was markedly reduced by p38 MAPK inhibition under l‐serine depletion, whereas p38 MAPK could be activated by 1‐deoxysphinganine, an atypical alanine‐derived sphingoid base that was found to accumulate in l‐serine‐depleted mouse embryonic fibroblasts. These observations provide persuasive evidence that when the external l‐serine supply is limited, l‐serine synthesized *de novo* in proliferating cells serves as a metabolic gatekeeper to maintain cell survival and the functions necessary for executing cell cycle progression.

**Database:**

Gene Expression Omnibus, accession number GSE55687.

**Abbreviations**
4E‐BP1: eukaryotic initiation factor 4E‐binding protein 1
AMPK: AMP‐activated protein kinase
Ccnd1: cyclin D1
Cdkn1a/p21: cyclin‐dependent kinase inhibitor 1a
doxSA: 1‐deoxysphinganine
HSAN1: hereditary sensory and autonomic neuropathy type I
HT: heterozygous
MEFs: mouse embryonic fibroblasts
Phgdh: d‐3‐phosphoglycerate dehydrogenase
Rb: retinoblastoma
SAPK/JNK: stress‐activated protein kinase/Jun amino‐terminal kinase
SA: sphinganine
SO: sphingosine
SPT: serine palmitoyl‐CoA transferase

l‐Serine (l‐Ser) plays versatile roles in metabolism as a necessary precursor for the synthesis of various biomolecules such as proteins, other nonessential amino acids, membrane lipids, and nucleotides, all of which are important for cell proliferation. Lockart and Eagle demonstrated in the late 1950s that l‐Ser supplementation greatly improves the growth of normal and cancer cells in culture 1. Recent studies have also shown that enhanced synthesis of l‐Ser promotes malignant properties, metabolic reprogramming, and resistance to oxidative stresses in some cancer cell lines 2, 3, 4. Our previous *in vivo* study demonstrated that severe l‐Ser deficiency in mice induced by targeted disruption of the d‐3‐phosphoglycerate dehydrogenase gene (*Phgdh*), which encodes an enzyme that catalyzes the first commitment step of the *de novo* l‐Ser synthetic pathway, results in a lethal phenotype after embryonic day (E)13.5 that is associated with brain malformation and overall growth retardation 5. Thus, there is an absolute necessity for *de novo*‐synthesized l‐Ser for embryonic viability and tissue growth.

We recently demonstrated that initiation of mRNA translation is entirely suppressed in the central nervous system of *Phgdh* KO embryos by a robustly induced eukaryotic initiation factor 4E‐binding protein 1 (4E‐BP1) 6. 4E‐BP1 is also induced in a MEF cell line established from *Phgdh* KO embryos under l‐Ser‐depleted conditions 6. Overexpression of a constitutively active 4E‐BP1 mutant in a rat embryonic cell line delays cell proliferation by inhibiting G1 progression 7. Taken together, these results suggest that l‐Ser deficiency deregulates cell cycle progression in normal cells, which may be a mechanism to arrest growth in *Phgdh* KO embryos. However, the mechanisms by which normal cells adapt to l‐Ser deficiency and by which reduced availability of l‐Ser affects the cell cycle machinery remain largely unexplored. Here, we used *Phgdh*‐deficient MEFs to show that p38 MAPK is activated by l‐Ser deficiency and mediates induction of cyclin‐dependent kinase inhibitor 1a (p21). This p38 MAPK activation participated in maintaining survival of KO‐MEFs in cellular environment with reduced l‐Ser availability, which seemed likely to be sensed by an atypical alanine‐derived sphingoid base 1‐deoxysphinganine (doxSA) generated and accumulated under l‐Ser‐depleted condition. Inhibition of p38 MAPK under l‐Ser depletion led to massive cell death in *Phgdh*‐deficient MEFs, indicating that the activation of p38 MAPK via doxSA is required for cells to survive l‐Ser deficiency.

## Materials and methods

### Cell culture

Spontaneously immortalized MEFs were established from individual E13.5 embryos of WT, *Phgdh* heterozygous (HT), and *Phgdh* KO genotypes and maintained as described 6. KO‐MEFs transduced retrovirally with the mouse Phgdh cDNA (KO‐MEF^+*Phgdh*^ cells) 8 and GFP (KO‐MEF^+*GFP*^ cells) were also used. The latter was prepared as follows. The GFP coding sequence from pCMX‐SAH/Y145F was subcloned into the retroviral expression vector pCX4bsr (GenBank ID: AB086384). The expression vector plasmid for *GFP* (pCX4‐GFP‐ires‐bsr) was introduced into the packaging cell line Platinum‐E 9 with the vectors pE‐eco and pGP (TaKaRa, Shiga, Japan) by using polyethyleneimine ‘Max’ (Polysciences, Warrington, PA, USA), and the transfected cells were cultured for 48 h. The supernatant was collected, filtered through a 0.45‐μm filter, and used as the retrovirus stock. KO‐MEFs were infected by mixing with the viral stock diluted 1 : 1 in fresh medium containing polybrene (Sigma‐Aldrich, St. Louis, MO, USA) and incubated overnight at 33 °C. The infection was repeated at 6 and 18 h after the initial infection. Then infected cells were selected in medium containing 10 μg·mL^−1^ Blasticidin S (Kaken Pharmaceutical, Tokyo, Japan) at 37 °C for 2–3 days.

Pooled clones of immortalized MEFs were used in this study. All cell lines were cultured as described 6. Briefly, cells were maintained in complete high‐glucose DMEM medium (Wako Pure Chemical Industries Ltd., Osaka, Japan) supplemented with 10% FBS (Gibco, Thermo Fisher Scientific, Waltham, MA USA), 4 mm glutamine (584 mg·L^−1^), and 10 μg·mL^−1^ gentamicin (Nacalai Tesque, Kyoto, Japan). To deprive MEFs of l‐Ser, the complete DMEM medium was replaced with Eagle's Minimum Essential medium with Earle's salts (EMEM; Wako Pure Chemical Industries Ltd.) supplemented with 1% FBS and 10 μg·mL^−1^ gentamicin, which contained all essential amino acids and l‐glutamine but did not include l‐Ala, l‐Asp, l‐Asn, l‐Cys, l‐Glu, l‐Gly, l‐Pro, and l‐Ser. This medium was used for the l‐Ser‐depleted condition, as it contained 4 μm l‐Ser, which was derived from the 1% FBS. When EMEM supplemented with 10% FBS was used, l‐Ser derived from FBS reached to ~ 40 μm. The l‐Ser‐supplemented condition was established by adding l‐Ser (final 400 μm) to EMEM supplemented with 1% FBS and 10 μg·mL^−1^ gentamicin. Amino acids were all purchased from Nacalai Tesque unless otherwise indicated. l‐Ala (Wako Pure Chemical Industries Ltd), l‐Asp, l‐Asn, l‐Cys, l‐cystine (l‐CySS), Gly (Wako Pure Chemical Industries Ltd), l‐Pro, d‐Ser (Sigma‐Aldrich), and l‐Ser (Sigma‐Aldrich) were individually prepared as a stock solution (200 mm). l‐Glu was prepared as a 40 mm stock solution. Amino acid stock solutions were individually added to EMEM supplemented with 1% FBS and 10 μg·mL^−1^ gentamicin to a final concentration of 400 μm to examine their effects on survival and/or gene expression in MEFs.

### Cell growth assay

Cells were seeded at 1.0–1.5 × 10^4^ cells per well in a 24‐well plate containing the complete DMEM medium and were incubated for 12 h. The medium was then changed to either the complete DMEM medium, EMEM with 10% FBS, or EMEM with 1% FBS with or without 400 μm l‐Ser, and the cells were cultured for the indicated time periods. Live cells were counted using either the Cell Counting Kit‐8 (Dojindo Laboratories, Kumamoto, Japan) or trypan blue exclusion. Half of the culture medium was replaced with fresh medium every 24 h.

#### BrdU labeling

KO‐MEFs were seeded at 0.5–1.0 × 10^4^ cells per well in a 96‐well clear‐bottom μClear‐Plate (Greiner Bio‐One, Frickenhausen, Germany) containing the complete DMEM medium and were incubated overnight (12–24 h). The medium was then changed to EMEM with or without 400 μm l‐Ser, and cells were incubated for 24 h. BrdU (Dako, Agilent Technologies, Santa Clara, CA, USA) was then added to a final concentration of 10 μm. After a 30‐min incubation, the medium was replaced with chilled 70% ethanol, and the plates were placed at −20 °C for 10 min, washed twice with DPBS, and then incubated at 37 °C for 30 min with 2 N HCl containing 0.7% Triton X‐100. Sodium tetraborate (0.1 m, pH 8.5) was added to neutralize the acid. Mouse anti‐BrdU (1 : 250 in DPBS containing 3% BSA and 0.1% Triton X‐100; Dako) was added, and the plates were incubated overnight at 4 °C. Rabbit anti‐mouse IgG labeled with Alexa Fluor 555 (1 : 1850; Molecular Probes, Thermo Fisher Scientific) diluted in DPBS containing 20 μg·mL^−1^ RNase was added, and the plates were incubated for 1 h at room temperature. Then cell nuclei were visualized with Hoechst 33342 (1 : 1000; Dojindo Laboratories). The images of Hoechst 33342 and Alexa Fluor 555 staining were acquired using an IN Cell Analyzer 1000 (GE Healthcare, Little Chalfont, Buckinghamshire, UK) using a 20× objective and 360‐nm (Hoechst 33342) and 535‐nm (Alexa Fluor 555) excitation filters, monitored through 460‐nm and 600‐nm emission filters, respectively, with a 61000v2 trichronic mirror as described previously 10. Images of Hoechst 33342 and Alexa Fluor 555 staining were analyzed by using the developer software (GE Healthcare). Images collected from 460‐nm and 600‐nm emission filters were used to define the nuclei, and the intensity of Alexa Fluor 555 fluorescence was evaluated in 4895 cells for the l‐Ser‐supplemented condition and 4708 cells for the l‐Ser‐depleted condition. The data collected with developer were applied to spotfire decisionsite client 8.2 software (GE Healthcare UK Ltd., Buckinghamshire, UK) to visualize the results.

### Flow cytometry

Cells were seeded at 0.8–4.0 × 10^5^ cells per 100‐mm dish containing the complete DMEM medium and were incubated for 24 h. The medium was then changed to EMEM with 1% FBS and either with or without 400 μm l‐Ser, and the cells were cultured for 24 h. Cells were trypsinized and centrifuged at 190 ***g*** for 5 min. Each pellet was suspended in 1 mL of DPBS and fixed with 2.5 mL of absolute ethanol (final concentration ~ 70%) and incubated overnight at −20 °C. Cells were centrifuged at 420 ***g*** for 5 min, and the pellet was resuspended in 300 μL of DPBS containing 50 μg·mL^−1^ propidium iodide (Dojindo Laboratories), 0.1 mg·mL^−1^ RNase A (Nacalai Tesque) and 0.05% TritonX‐100. The resulting cell suspension was incubated for 40 min at 37 °C and then was centrifuged at 420 ***g*** for 5 min after the addition of 3 mL of DPBS. The pellet was resuspended in 500 μL of DPBS, and the cell suspension was applied to a LSR II flow cytometer (BD, Franklin Lakes, NJ, USA) to analyze the cell cycle.

### Microarray analysis

Gene expression microarray analysis was performed as described 11. In brief, total RNA was extracted from KO‐MEFs after a 6‐h incubation under l‐Ser‐depleted or ‐supplemented conditions and was purified using the RiboPure kit (Thermo Fisher Scientific). The integrity of the RNA samples was assessed by formaldehyde agarose gel electrophoresis. Samples with an absorbance ratio > 1.9 (i.e., 260 nm/280 nm ratio) were used for amplification and labeling for microarray chip hybridization. Total RNA (250 ng) was used to generate biotin‐labeled cDNA using the GeneChip 3′ IVT Express Kit (Affymetrix, Santa Clara, CA, USA). The biotin‐labeled amplified cDNA was fragmented and hybridized to Mouse Genome 430 2.0 GeneChip arrays (Affymetrix) for 16 h at 45 °C. After washing, the arrays were scanned with a GeneChipScanner (Affymetrix), and the scans were processed using the genesuite software (Affymetrix). Three biological replicates for each treatment were directly compared. Raw microarray data were transferred to genespring 8.0 software (Agilent Technologies), and the fluorescence intensity of each spot on the microarray was normalized to its median value. To screen for differentially expressed genes between the l‐Ser‐depleted and ‐supplemented conditions, the normalized data were filtered based on the following criteria: (a) scaled intensity > 100 under at least one condition; (b) false discovery rate, *q* < 0.01; and (c) absolute value of fold change (l‐Ser‐depleted condition/l‐Ser‐supplemented) > 2.0 or < 0.5. A list of 941 probe sets corresponding to differentially expressed genes was generated using these criteria. Significantly represented biological themes and functional groups in the differentially expressed gene list were analyzed using the web‐based expression analysis program Ingenuity Pathways Analysis (http://www.ingenuity.com). The microarray data have been submitted to the Gene Expression Omnibus, accession number GSE55687.

### Western blot analysis

Protein extracts were prepared as follows. After appropriate incubation periods, cells were washed twice with cold DPBS, scraped and homogenized with a 26‐gauge needle in cold RIPA Buffer (25 mm Tris‐HCl, pH 7.6; 150 mm NaCl; 1% Nonidet P‐40; 1% sodium deoxycholate; 0.1% SDS; phosphatase inhibitor cocktail; and protease inhibitor cocktail; Nacalai Tesque). The homogenates were centrifuged at 20 400 ***g*** for 30 min at 4 °C, and the supernatants were collected as MEF protein samples. Protein extracts were fractionated by SDS/PAGE and electroblotted onto a PVDF membrane (Pall Corporation, Port Washington, NY, USA). Blotted proteins were probed with the following primary antibodies: anti‐Phgdh (rabbit polyclonal, 1 : 2000 5, anti‐p21 (rabbit polyclonal, 1 : 50; Abcam, Cambridge, UK), anti‐cyclin D1 (Ccnd1) (mouse monoclonal, 1 : 1000; Calbiochem, Merck, Darmstadt, Germany), anti‐retinoblastoma protein (Rb) (rabbit polyclonal, 1 : 1000; Cell Signaling Technology, Danvers, MA, USA), anti‐phospho‐Rb (Ser780; rabbit polyclonal, 1 : 500; Cell Signaling Technology), anti‐phospho‐p38 MAPK (Tyr182; rabbit polyclonal, 1 : 200; New England BioLabs, Ipswich, MA, USA), anti‐p38 MAPK (rabbit polyclonal, 1 : 2000; New England BioLabs), anti‐stress‐activated protein kinase (SAPK)/Jun N‐terminal kinase (JNK) (rabbit polyclonal, 1 : 500; New England BioLabs), anti‐phospho‐SAPK/JNK (Thr183/Tyr185; rabbit monoclonal, 1 : 500; Cell Signaling Technology), anti‐p44/42 MAPK (Erk1/2) (rabbit polyclonal, 1 : 1000; Cell Signaling Technology), anti‐phospho‐Erk1/2 (Thr202/Tyr204; rabbit polyclonal, 1 : 1000; Cell Signaling Technology), anti‐AMP‐activated protein kinase (AMPK)α (rabbit polyclonal, 1 : 1000; Cell Signaling Technology), anti‐phospho‐AMPKα (Thr172; rabbit polyclonal, 1 : 500; Cell Signaling Technology), and anti‐Gapdh (mouse monoclonal, 1 : 50 000; Chemicon, Merck, Darmstadt, Germany). Antibodies against phospho‐p38 MAPK, phospho‐SAPK/JNK, phospho‐Erk1/2, and phospho‐AMPKα recognize active forms of these kinases. Bound antibodies were visualized with the Pierce SuperSignal West Pico chemiluminescence detection system (Thermo Fisher Scientific) after being incubated with the appropriate horseradish peroxidase‐conjugated secondary antibodies (Cell Signaling Technology and New England BioLabs). The chemiluminescent signal was detected as described 6. The Gapdh signal was used as an internal standard to normalize the respective p21 and Ccnd1 data. Phosphorylation of Rb was estimated by the ratio of the amount of the Ser780‐phosphorylated form to the amount of total Rb from the same blot. Phosphorylation of p38 MAPK, SAPK/JNK, Erk1/2, and AMPKα were estimated by the ratio of the amount of the Tyr182‐phosphorylated form to the amount of total p38 MAPK, Thr183/Tyr185‐phosphorylated form to the amount of total SAPK/JNK, Thr202/Tyr204‐phosphorylated form to the amount of total Erk1/2, and Thr172‐phosphorylated form to the amount of total AMPKα, respectively, from each blot.

### Quantitative real‐time PCR

Total RNA from MEFs was extracted using either the RiboPure kit or the High Pure RNA Isolation kit (Roche Diagnostics, Basel, Switzerland), and 1 μg of total RNA was used for cDNA synthesis. Quantitative Real‐time PCR (qPCR) was performed with a qPCR System Mx3000P (Agilent Technologies) using the THUNDERBIRD SYBR qPCR Mix (TOYOBO, Osaka, Japan) as described 6. Primers used were *p21* forward, 5′‐GTGGCCTTGTCGCTGTCTTG‐3′, and reverse, 5′‐AAGAGGCCTCCTGACCCACA‐3′; *Ccnd1* forward, 5′‐CATTCCCTTGACTGCCGAGA‐3′, and reverse, 5′‐TTGCGGATGGTCTGCTTGTT‐3′; and *Gapdh* forward, 5′‐ACTCCCACTCTTCCACCTTCG‐3′, and reverse, 5′‐ATGTAGGCCATGAGGTCCACC‐3′. Expression of each PCR product was normalized to the *Gapdh* mRNA.

### Inhibitor treatments

The p38 MAPK inhibitor SB202190 HCl and the SAPK/JNK inhibitor SP600125 were purchased from Wako Pure Chemical Industries Inc. KO‐MEFs were cultured in the complete DMEM medium for 20 h, and then SB202190 (3 μm final) or SP600125 (25 μm final) was added to the culture medium. After a 1‐h incubation with the inhibitor, the culture medium was changed to EMEM containing 1% FBS in either the presence or absence of the inhibitor. After 24 h in the new medium, cell lysates were prepared, and protein expression was analyzed using western blot analysis as described 6. Total RNA was extracted after a 6‐h incubation under the culture conditions mentioned above, cDNA was synthesized, and *p21* mRNA was measured by qPCR as described 6. Effects of SB202190 on cell growth were measured after 48 h in the culture conditions mentioned above. The number of viable cells in each treatment was determined using trypan blue exclusion.

KO‐MEFs were cultured in the complete DMEM medium for 20 h, and then cells were incubated with the complete DMEM medium containing 0.1, 1, or 2 μm of MG132 (Merck) for 1 h prior to l‐Ser depletion with the same concentration of MG132 in EMEM containing 1% FBS for 6 h. Cell lysates were then prepared, and Ccnd1, phospho‐Rb, and Rb were analyzed by western blotting as described above.

### Sphingolipid treatments

doxSA and sphinganine (SA) were purchased from Avanti Polar Lipids (Alabaster, AL, USA). Sphingosine (SO; 10 mg·mL^−1^ in isopropanol) was from Matreya, LLC (State College, PA, USA). SA was prepared as a 50 mm stock solution made up in ethanol. KO‐MEFs were cultured in the complete DMEM medium for 20 h, and then the medium was replaced with l‐Ser‐supplemented EMEM containing 1% FBS and doxSA (0.1, 1, or 5 μm), SA (5 μm), or SO (5 μm) for 6 or 24 h. Total RNA was then extracted using the High Pure RNA Isolation kit, cDNA was synthesized, and *p21* mRNA was measured by qPCR as described above. Protein extracts were prepared, and western blot analysis was performed as described 6.

### Statistical analysis

Differences between two groups were examined with the Student's *t*‐test. Differences between more than two groups were analyzed with one‐way analysis of variance followed by Dunnett's *post hoc* test; *P*‐values < 0.05 were considered significant. Values presented are the mean and standard error of the mean (SEM) for three replicate measurements unless otherwise indicated. All statistics were performed using kaleidagraph 4.0 (Synergy Software, Reading, PA, USA).

## Results

### l‐Ser deficiency‐induced growth arrest in *Phgdh* KO‐MEFs

**Figure 1:**
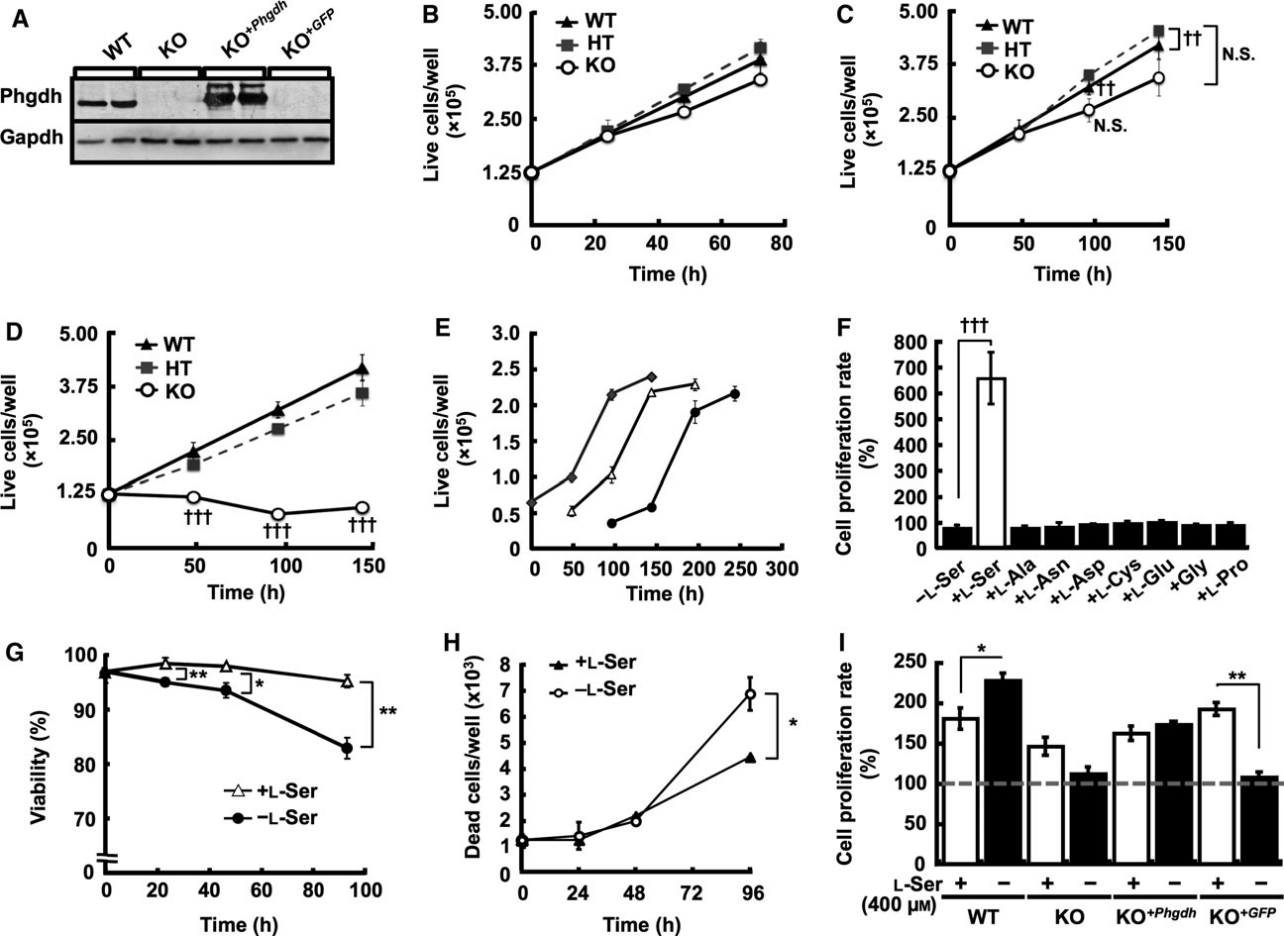
External l‐Ser is necessary for proliferation of KO‐MEFs. (A) The indicated cell lines were cultured in DMEM containing 10% FBS for 24 h, and Phgdh expression was examined by western blot analysis. (B–D) Live cell numbers of WT‐, HT‐, and KO‐MEFs were determined after incubation in complete DMEM medium containing 10% FBS for 0, 24, 48, and 72 h (B); EMEM containing 10% FBS for 0, 48, 96, and 144 h (Dunnett's *post hoc* test, ^††^ *P* < 0.005 compared with corresponding WT‐MEFs) (C); or EMEM containing 1% FBS (l‐Ser depletion) for 0, 48, 96, and 144 h (Dunnett's *post hoc* test, ^†††^ *P* < 0.0005 compared with corresponding WT‐MEFs) (D). (E) KO‐MEFs were cultured under the l‐Ser‐depleted condition for 0 (♦), 48 (▵), and 96 h (●), and then the medium was replaced with l‐Ser‐supplemented medium, and live cells were counted after culturing for the indicated time. (F) Live KO‐MEFs were counted after a 48‐h incubation in EMEM containing 1% FBS supplemented with the indicated nonessential amino acids (400 μm). ^†††^Dunnett's *post hoc* test, *P* < 0.0005. (G) KO‐MEFs were cultured under the l‐Ser‐depleted or ‐supplemented condition for 0, 24, 48, and 96 h. Live and dead cells were counted at each time point using trypan blue exclusion to calculate cell viability (Student's *t*‐test, *P < 0.05, **P < 0.005). (H) KO‐MEFs were cultured under the l‐Ser‐depleted or ‐supplemented condition for 0, 24, 48, and 96 h, and the number of dead cells was determined by trypan blue exclusion. Student's *t*‐test, *P < 0.05. (I) The indicated cell lines were cultured under the l‐Ser‐depleted or ‐supplemented condition for 24 h, and live cells were counted. Student's *t*‐test, *P < 0.05, **P < 0.005.

In this study, several MEF lines were used to define the molecular mechanisms underlying the effect of l‐Ser deficiency on the core cell cycle machinery. First, we examined Phgdh protein levels in those MEF lines by western blot analysis, and confirmed the cell lines lacking functional *Phgdh* did not express Phgdh protein (Fig. 1A). To discern if there is a mechanistic link between cell cycle progression and l‐Ser availability, we characterized the growth properties of spontaneously immortalized MEFs lacking *Phgdh* (KO‐MEFs) under three different culture conditions. KO‐MEFs grew similarly to WT‐ and HT‐MEFs in complete DMEM containing all amino acids supplemented with 10% FBS (Fig. 1B) and in EMEM supplemented with 10% FBS but lacking l‐Ala, l‐Asn, l‐Asp, l‐Cys, l‐Glu, Gly, l‐Pro, and l‐Ser (Fig. 1C). We next examined the effect of lower FBS supplementation (1%) on cell growth, because the 10% FBS contained ~ 40 μm l‐Ser. Unlike WT‐ and HT‐MEFs, KO‐MEFs exhibited cell growth arrest under this 1% FBS–EMEM condition (Fig. 1D). We previously established that the intracellular free l‐Ser and Gly contents are significantly reduced in KO‐MEFs maintained in the 1% FBS–EMEM condition compared with those supplemented with 400 μm l‐Ser 6. Proliferation of KO‐MEFs was completely restored by addition of l‐Ser to the medium, even 96 h after switching to EMEM containing 1% FBS (Fig. 1E). KO‐MEFs could grow only in the presence of external l‐Ser, but not Gly or other non‐essential amino acids (Fig. 1F). Furthermore, a gradual decrease in cell viability (Fig. 1G) and a significant increase in the number of dead cells over time (Fig. 1H) were evident in KO‐MEFs maintained in EMEM containing 1% FBS, both of which were prevented by addition of l‐Ser to the medium (Fig. 1G,H). These observations suggested that the reduced availability of l‐Ser was responsible for the cell proliferation arrest and death of KO‐MEFs. Indeed, KO‐MEFs transduced with mouse *Phgdh* cDNA (KO‐MEF^+*Phgdh*^) and WT‐MEFs grew in EMEM containing 1% FBS, whereas KO‐MEFs transduced with GFP (KO‐MEF^+*GFP*^), which did not have functional Phgdh, did not grow without l‐Ser supplementation (Fig. 1I).

### Induction of p21 in KO‐MEFs

**Figure 2:**
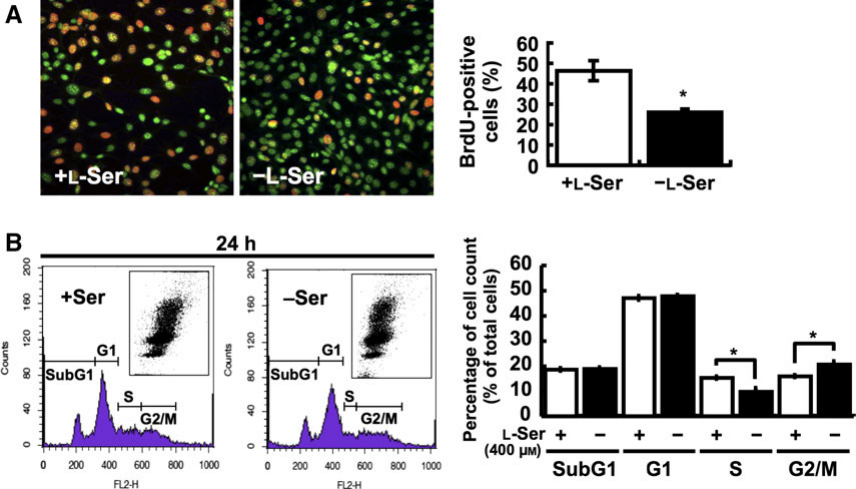
l‐Ser depletion elicits cell cycle arrest in KO‐MEFs. (A) BrdU‐positive (orange/yellow) cells were counted after a 24‐h incubation under the l‐Ser‐supplemented (+l‐Ser) or ‐depleted (−l‐Ser) condition. Nuclei were stained with Hoechst (green). Student's *t*‐test, *P < 0.05. (B) KO‐MEFs were cultured under the l‐Ser‐depleted or ‐supplemented condition for 24 h, and cell cycle analysis was performed by flow cytometry. Representative dot blot, flow cytometric data, and statistical data are shown. Student's *t*‐test, *P < 0.05; *n* = 5 each.

A BrdU incorporation assay revealed that cell cycle progression was affected in KO‐MEFs grown in EMEM containing 1% FBS (hereafter designated as the l‐Ser‐depleted condition, 4 μm l‐Ser). The proportion of BrdU‐positive KO‐MEFs indicating the number of cell in the S phase is lower under the l‐Ser‐depleted condition than under the l‐Ser‐supplemented condition (Fig. 2A). Flow cytometric analysis of KO‐MEFs also demonstrated a decrease in S‐phase fraction and a simultaneous increase in G2/M‐phase fraction under l‐Ser depletion compared with those under l‐Ser supplementation (Fig. 2B).

**Figure 3:**
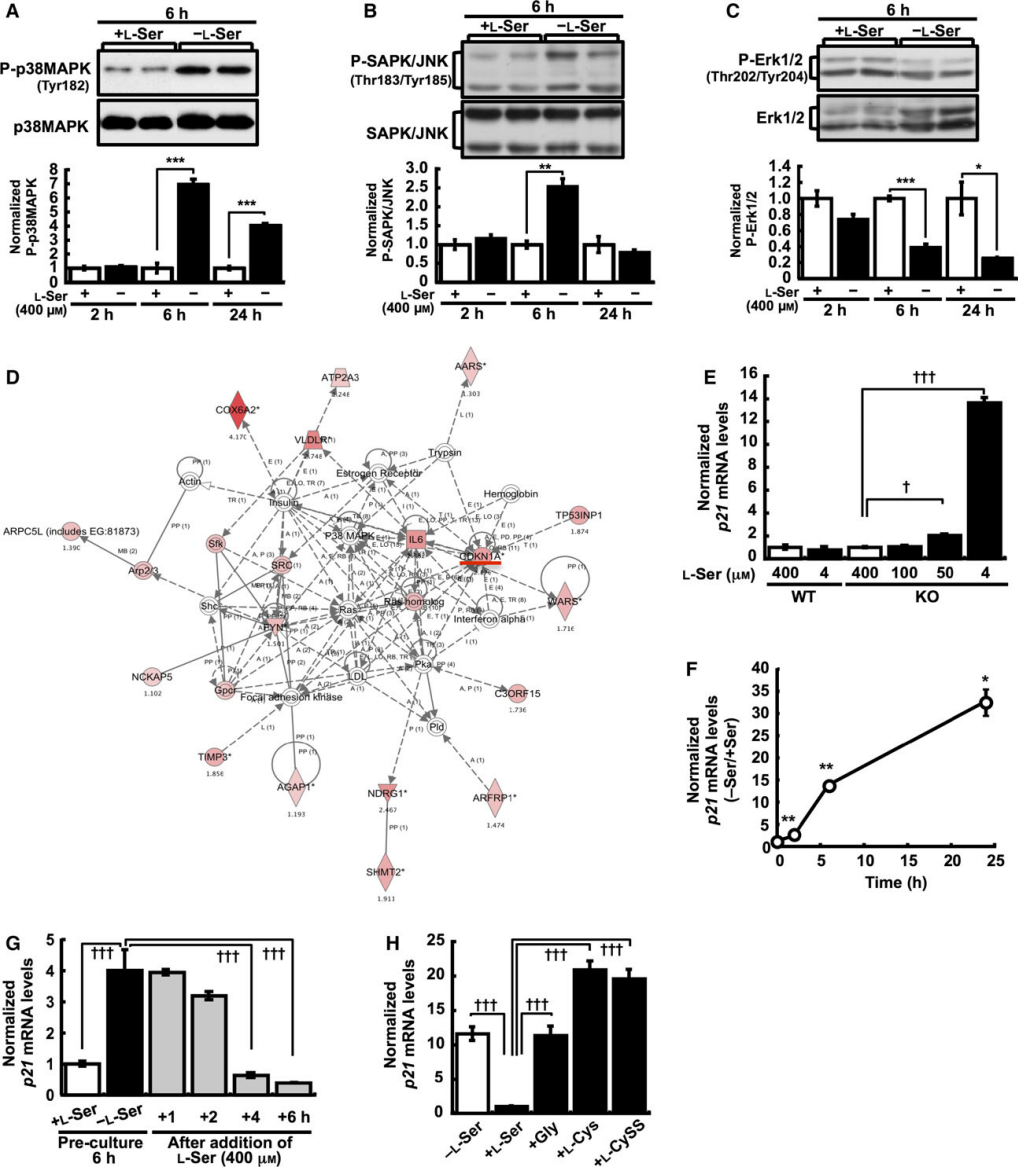
l‐Ser deficiency activates MAPK and p21 expression in KO‐MEFs. **(**A–C) KO‐MEFs were cultured for the indicated times under the l‐Ser‐depleted or ‐supplemented condition, and the phosphorylation (P) levels at Tyr182 of p38 MAPK (A), Thr183/Tyr185 of SAPK/JNK (B), and Thr202/Tyr204 of Erk1/2 (C) were determined after normalization to the amount of corresponding total kinase on the same western blot (p38 MAPK,* n =* 3 each for 2 and 6 h; *n* = 4 for 24 h; SAPK/JNK and Erk1/2, *n =* 4 for each time point). Representative western blots of two samples from each condition are shown. (D) Molecular networks involving *p21* that were significantly up‐regulated in KO‐MEFs under l‐Ser depletion. The networks are displayed graphically as nodes (genes/proteins) and lines (the biological relationships between the nodes). The intensity of the node color (red) indicates the degree of up‐regulation, and the node shapes represent functional classes. The line/arrow labels indicate the nature of the relationship between the nodes as follows: A, activation; B, binding; E, expression; I, inhibition; LO, localization; P, phosphorylation/dephosphorylation; PD, protein‐DNA binding; PR, protein‐mRNA binding; PP, protein–protein binding; T, transcription. Solid lines indicate direct interactions, and dashed lines indicate indirect interactions. Lines without a label represent binding only. Red line indicates p21. (E) WT‐ and KO‐MEFs were cultured for 6 h in l‐Ser‐depleted (4 μm) or ‐supplemented (50, 100, 400 μm) EMEM, and *p21* mRNA levels were measured. (F) Time course of *p21* expression after l‐Ser depletion in KO‐MEFs. qPCR was performed using samples collected at 2, 6, and 24 h after incubation under the l‐Ser‐depleted or ‐supplemented condition. (G) KO‐MEFs were cultured under the l‐Ser‐depleted or ‐supplemented condition for 6 h, and then l‐Ser‐depleted medium was replaced with l‐Ser‐supplemented medium, and *p21* mRNA was measured after culturing for the indicated times. (H) KO‐MEFs were cultured under the l‐Ser‐depleted or l‐Ser‐, Gly‐, l‐Cys‐, or l‐CySS‐supplemented (final, 400 μm each) condition for 6 h, and *p21* mRNA levels were evaluated. Student's *t*‐test, *P < 0.05, **P < 0.005, ***P < 0.0005; Dunnett's *post hoc* test, ^†^ *P* < 0.05, ^†††^ *P* < 0.0005.

In addition to retarded cell cycle progression, we recently demonstrated that lipid body formation occurred in KO‐MEFs cultured under l‐Ser‐depleted condition 8. It made us presume that these changes may elicit a kind of stress to cells affecting gene/protein expression in l‐Ser‐depleted KO‐MEFs. Thus, to identify molecules affected by l‐Ser‐depletion and the molecular mechanism underlying the cell growth arrest, we performed preliminary semiquantitative analysis of phosphopeptides using mass spectrometry as described 12 and gene expression microarray analysis of KO‐MEFs. As a result, we found that l‐Ser depletion promoted phosphorylation of peptide fragments derived from p38 MAPK and other proteins in KO‐MEFs (T. Sayano, T. Nabetani, Y. Hirabayashi & S. Furuya, unpublished observation), suggesting that p38 MAPK is modulated by l‐Ser deficiency, and might be other stress‐related kinases as well. We thus examined whether p38 MAPK, SAPK/JNK, or p44/42 MAPK (Erk1/2) were activated by l‐Ser deficiency. We observed transient but significant increases in the proportions of phosphorylated p38 MAPK (Fig. 3A) and SAPK/JNK (Fig. 3B) in l‐Ser‐depleted KO‐MEFs within 6 h, whereas the proportion of phosphorylated Erk1/2 was reduced (Fig. 3C), indicating that l‐Ser deficiency up‐regulates stress‐responsive MAPK pathways.

In parallel, we found that cell cycle‐related factors and processes were markedly affected under the l‐Ser‐depleted condition by gene expression microarray analysis (Table 1). Among the genes involved in regulation of the cell cycle, the cyclin‐dependent kinase inhibitor 1a (*Cdkn1a*, also known as *p21*), but not other CDK inhibitors, was significantly up‐regulated in KO‐MEFs under l‐Ser depletion (Fig. 3D). qPCR analysis confirmed that the levels of *p21* mRNA dramatically increased in KO‐MEFs, but not in WT‐MEFs, as l‐Ser concentrations in the culture medium decreased (Fig. 3E) and over time (Fig. 3F). Later addition of l‐Ser into the culture medium completely suppressed this elevation of *p21* mRNA (Fig. 3G), whereas addition of Gly, Cys, or Cyss failed to lower *p21* mRNA levels (Fig. 3H). These observations indicated that *p21* mRNA was principally induced by reduced l‐Ser availability in KO‐MEFs.

**Table 1:** l‐Ser‐depletion‐induced biological functions in KO‐MEFs determined by gene expression microarray.^a^

Bio functions	*P*‐value (range)	Numbers of molecules belonged to each category
Molecular and cellular functions		
Cell cycle	1.63E‐04 to 4.96E‐02	55
Amino acid metabolism	3.97E‐04 to 3.59E‐02	10
Molecular transport	3.97E‐04 to 4.23E‐02	21
Small molecule biochemistry	3.97E‐04 to 3.59E‐02	20
Cellular growth and proliferation	6.69E‐04 to 4.68E‐02	94
Physiological system development and function		
Embryonic development	1.63E‐04 to 2.78E‐02	18
Hair and skin development and function	6.40E‐04 to 4.23E‐02	12
Organ development	6.40E‐04 to 4.91E‐02	20
Renal and urological system development and function	7.69E‐04 to 4.62E‐03	5
Diseases and disorders		
Infection mechanism	4.45E‐03 to 4.16E‐02	10
Infectious disease	4.45E‐03 to 2.78E‐02	2
Cancer	7.28E‐03 to 3.35E‐02	19
Organismal injury and abnormalities	9.35E‐03 to 2.99E‐02	26
Developmental disorder	1.07E‐03 to 2.78E‐02	4

a Genes that were significantly and at least 2.0‐fold up‐regulated in l‐Ser‐depleted KO‐MEFs compared to l‐Ser‐supplemented KO‐MEFs were categorized by biological functions using Ingenuity Pathways Analysis.

### p38 MAPK mediates p21 induction

**Figure 4:**
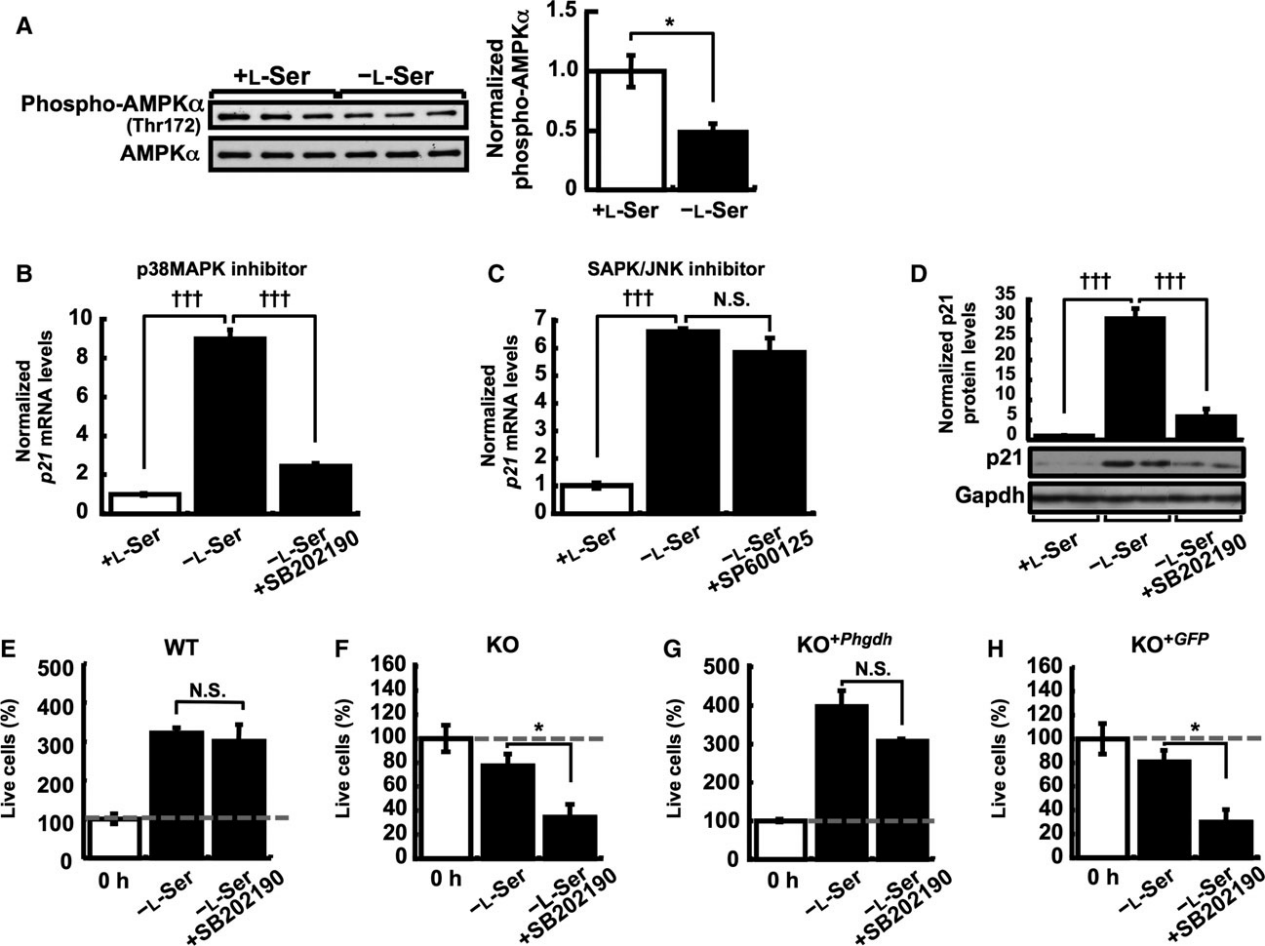
Activation of p38 MAPK is necessary for cell survival in KO‐MEFs under l‐Ser depletion. (A) KO‐MEFs were cultured for 6 h under the l‐Ser‐depleted or ‐supplemented condition, and phosphorylation at Thr172 of AMPKα was examined by western blot and normalized to the total AMPKα on the same blot for quantification. (B and C) *p21* mRNA levels in KO‐MEFs were determined after a 6‐h incubation under the l‐Ser‐supplemented condition or the l‐Ser‐depleted condition in the presence or absence of 3 μm SB202190 (B) or 25 μm SP600125 (C). (D) p21 protein levels in KO‐MEFs were determined after a 24‐h incubation under the l‐Ser‐supplemented condition or the l‐Ser‐depleted condition in the presence or absence of 3 μm SB202190 (*n =* 4 each). Representative western blots show two samples from each treatment. Gapdh was used as an internal standard, and p21 expression was normalized to that of Gapdh. (E–H) The number of live cells was measured in WT‐MEF (E), KO‐MEF (F), KO‐MEF ^+*Phgdh*^ (G), and KO‐MEF ^+^ ^*GFP*^ (H) cell lines after a 48‐h incubation under the l‐Ser‐depleted condition in the presence or absence of 3 μm SB202190. Student's *t*‐test, *P < 0.05; Dunnett's *post hoc* test, ^†††^ *P* < 0.0005; N.S., not significant.

p21 is a critical mediator of p53‐dependent cell cycle arrest at G1‐ and G2/M‐phases in response to metabolic perturbation of nucleotide synthesis and DNA damage 13. Previous studies have reported that p21 is transiently induced simultaneously with the activation of the AMPK‐p53 cascade by nutritional depletion of either glucose or l‐Ser/Gly in MEFs and certain cancer cell lines, and the resultant cell cycle arrest contributes to cell survival 4, 14. AMPK activation and subsequent phosphorylation of p53 at Ser15 (Ser18 in mouse) are required for *p21* induction in MEFs that results from glucose limitation 14. Unlike glucose limitation, we found that phosphorylation at Thr172 of AMPKα, which is associated with activation 15, actually decreased under l‐Ser depletion in KO‐MEFs (Fig. 4A).

We then tested whether p38 MAPK or SAPK/JNK regulates the *p21* induction that was caused by l‐Ser deficiency. The l‐Ser deficiency‐induced elevation in *p21* mRNA was greatly suppressed when l‐Ser‐depleted KO‐MEFs were simultaneously treated with the p38 MAPK inhibitor SB202190 HCl (hereafter referred to as SB202190), but not when treated with the SAPK/JNK inhibitor SP600125 (Fig. 4B,C). Similar suppression of p21 protein levels following l‐Ser depletion in the presence of SB202190 was also observed (Fig. 4D).

To better understand the pathophysiological significance of p38 MAPK activation by l‐Ser deficiency, we next examined the effect of SB202190 treatment on cell proliferation and survival of WT‐MEF, KO‐MEF, KO‐MEF^+*Phgdh*^, and KO‐MEF^+*GFP*^ cells (Fig. 4E–H). Treatment with SB202190 markedly reduced the number of viable KO‐MEF and KO‐MEF^+*GFP*^ cells, whereas WT‐MEF and KO‐MEF^+*Phgdh*^ cells did not show a significant decline in live cells and continue proliferation (Fig. 4E,G). These observations provide evidence that p38 MAPK activation triggered by l‐Ser deficiency is an adaptive response necessary for cell survival of KO‐MEFs lacking functional *Phgdh*.

### Activation of the doxSA–p38 MAPK–p21 axis by l‐Ser deficiency

**Figure 5:**
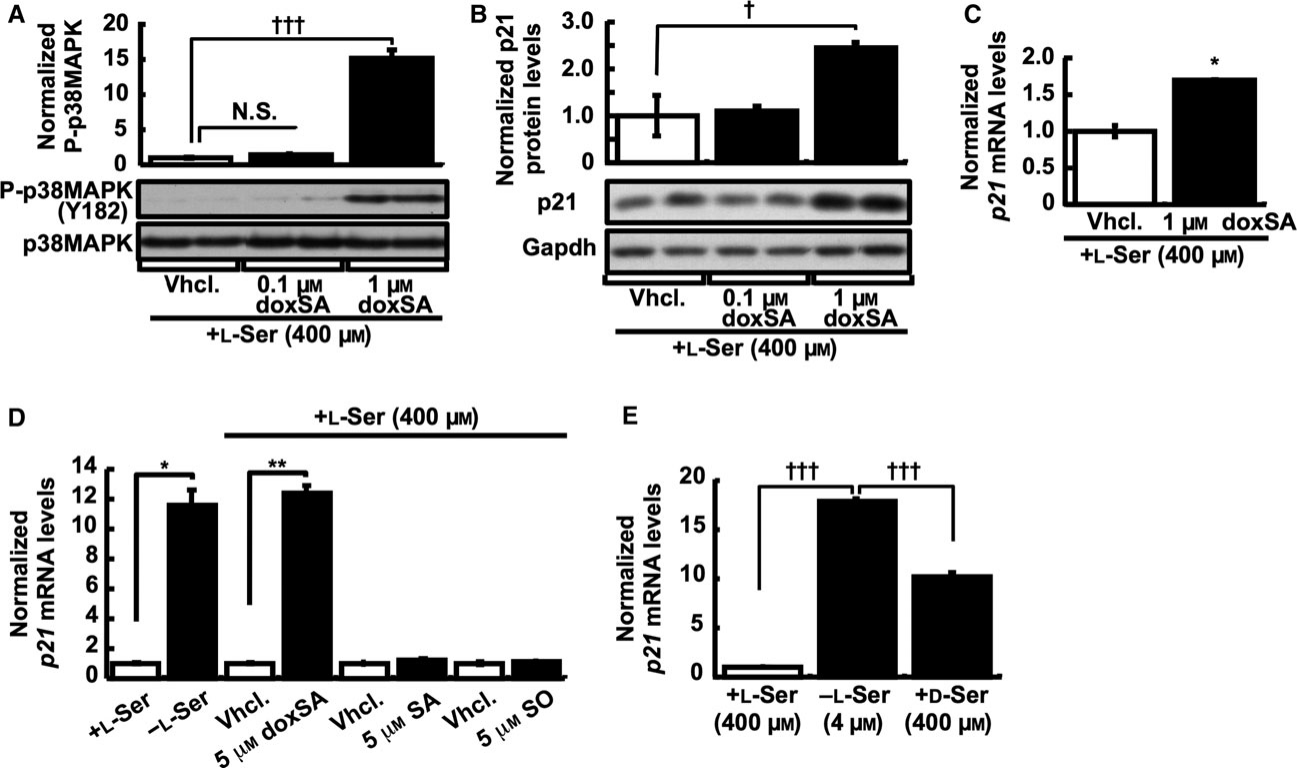
doxSA induces p38 MAPK and p21 expression in the presence of l‐Ser. **(**A) The phosphorylated and total p38 MAPK levels were detected in KO‐MEFs after treatment with doxSA for 24 h in the presence of l‐Ser. Dunnett's *post hoc* test, ^†††^ *P* < 0.0005; N.S., not significant. Representative western blots show two samples for each treatment. (B and C) KO‐MEFs were cultured for 24 h in the presence of 400 μm l‐Ser and either vehicle alone (Vhcl.) or vehicle with doxSA, and then p21 protein (B) (Dunnett's *post hoc* test, ^†^ *P* < 0.05) and mRNA (C) (Student's *t*‐test, *P < 0.05) levels were determined. Gapdh was used as an internal standard, and p21 expression was normalized to that of Gapdh. Representative western blots show two samples for each treatment. (D) *p21* mRNA levels were determined in KO‐MEFs maintained for 6 h with 5 μm doxSA, SA, or SO in the presence of 400 μm l‐Ser (Student's *t*‐test, *P < 0.05, **P < 0.005). (E) *p21* mRNA levels were determined in KO‐MEFs maintained for 24 h under the l‐Ser‐depleted, l‐Ser‐supplemented, or d‐Ser‐supplemented (400 μm) condition. Dunnett's *post hoc* test, ^†††^ *P* < 0.0005.

We next sought to identify factors that could regulate p38 MAPK in KO‐MEFs under l‐Ser depletion. The synthesis of sphingolipids is one of the major metabolic pathways that involve l‐Ser 16. Indeed, *Phgdh* KO tissues exhibit marked decreases in phosphatidylserine and glycosphingolipids 5. Our recent studies using a novel lipidomics approach based on mass spectrometry revealed that the loss of *Phgdh* promotes accumulation of an unusual molecular species of sphingolipid, 1‐deoxysphinganine (doxSA), also known as spisulosine/ES‐285, in embryonic tissues of *Phgdh* KO mice (A. H. Merrill & S. Bandyopadhyay, personal communication) and in KO‐MEFs depleted of l‐Ser, in which the doxSA level reached to ~ 0.6 pmol/10^6^ cells for 24 h 8. Spisulosine/ES‐285 treatment inhibits cell proliferation 17 via the disassembly of actin stress fibers 18 and leads to caspase‐3 activation and apoptosis 19 in various cancer cell lines. We therefore examined whether doxSA affects the expression of p38 MAPK and p21 in our system. KO‐MEFs were treated with doxSA in the presence of l‐Ser in the culture medium to examine the sole effect of doxSA, and levels of p38 MAPK protein and p21 mRNA and protein were determined. As we predicted, phosphorylation of p38 MAPK was enhanced by the addition of 1 μm doxSA to the culture medium even in the presence of 400 μm l‐Ser (Fig. 5A), which also elicited up‐regulation of p21 both protein and mRNA levels (Fig. 5B,C). Unlike doxSA, addition of Sphinganine (SA) and Sphingosine (SO), native metabolites of the ceramide synthesis and degradation pathways, respectively, had no effect on the expression of *p21* (Fig. 5D).

We presumed that doxSA could be formed by serine palmitoyl‐CoA transferase (SPT) catalyzing condensation of palmitoyl‐CoA with l‐Ala instead of l‐Ser during l‐Ser deficiency. Indeed, we have recently found that elevated doxSA levels in KO‐MEFs that are induced by l‐Ser deficiency are drastically decreased by d‐Ser which inhibits the catalytic activity of SPT 20, leading to the suppression of doxSA production by ~ 60% 8. When KO‐MEFs were treated with d‐Ser, a significant but incomplete (> 40%) reduction in *p21* mRNA was observed as compared with the l‐Ser‐depleted condition without d‐Ser (Fig. 5E), suggesting that doxSA generated via SPT directs up‐regulation of *p21* expression to some extent under l‐Ser depletion. This result implies the existence of doxSA–p38 MAPK–p21 axis when the external l‐Ser supply is limited.

In addition to p21 induction through p38 MAPK activation, we found that l‐Ser deficiency leads to alterations in cell cycle machinery, which was a marked reduction in cyclin D1 (Ccnd1) associated with diminished phosphorylation of retinoblastoma (Rb) protein. In response to mitotic stimuli, D‐type cyclins (Ccnd 1, 2, and 3) form a complex with cyclin‐dependent kinase (CDK) 4 or CDK6 and thereby phosphorylate Rb at Ser780, which is a necessary step for passage through the G1/S boundary 21, 22. CDK4/6‐bound or free Ccnd1 has an inherent instability and rapid turnover rate 23. We found that l‐Ser deficiency affected Ccnd1 and the phosphorylation status of Rb. Ccnd1 levels were greatly reduced in KO‐MEFs after 6 and 24 h of l‐Ser deficiency (Fig. S1A). Reduction in Ccnd1 under the l‐Ser‐depleted condition was also seen in KO‐MEF^+*GFP*^ cells, but not in WT‐MEF and KO‐MEF^+*Phgdh*^ cells (Fig. S1B), indicating that the primary cause of Ccnd1 reduction is l‐Ser deficiency that is due to the loss of *Phgdh*. In parallel with the decrease in Ccnd1, the ratio of phosphorylated Rb to total Rb was markedly decreased in KO‐MEFs after 6 and 24 h of l‐Ser depletion (Fig. S1C), which was also due to the lack of *Phgdh* (Fig. S1D). Taken together, these observations strongly suggest that l‐Ser deficiency leads to diminished levels of Ccnd1 and phosphorylated Rb, both of which likely contribute substantially to the cell cycle arrest in KO‐MEFs. It should be noted that decreases in Ccnd1 and phosphorylated Rb were not regulated by p38 MAPK (Fig. S1E,F) and doxSA (Fig. S1G,H). Although depletion of nonessential amino acids can suppress cell cycle progression via repression of *Ccnd1* transcription in hepatocytes 24, *Ccnd1* mRNA was not down‐regulated but rather was up‐regulated by l‐Ser deficiency in KO‐MEFs (Fig. S1I). Consistent with previous findings of ubiquitination and proteasomal degradation of Ccnd1 23, treatment of l‐Ser‐depleted KO‐MEFs with the protease inhibitor MG132 prevented both the reduction in Ccnd1 (Fig. S1J) and phosphorylation of Rb (Fig. S1K), suggesting that proteasome‐dependent degradation accounts for the marked decrease in Ccnd1 and consequent hypophosphorylation of Rb.

From the data obtained in this study and our previous studies, it is suggested that l‐Ser deficiency triggers activation of multiple cascades to adapt to and survive in l‐Ser starved environment, which include p38 MAPK activation through doxSA production as well as the GCN2‐eIF2α‐ATF4 pathway, a cascade elicited by amino acid deprivation, which was activated by reduced availability of l‐Ser in KO‐MEFs 6. Here, we present a schematic overview of the pathways activated by l‐Ser‐depleted conditions and propose a mechanism for the l‐Ser deficiency cascade (Fig. S2).

## Discussion

This study demonstrated that l‐Ser deficiency restricts cell cycle progression in embryonic fibroblasts via two independent mechanisms: the activation of the doxSA–p38 MAPK–p21 axis and the loss of the core cell cycle machinery components Ccnd1 and phosphorylated Rb. Induction of p21 was regulated primarily by p38 MAPK in KO‐MEFs, which appears to be an adaptive response to reduced availability of l‐Ser, because inhibition of p38 MAPK led to decreased viability in MEFs lacking *Phgdh*.

We identified doxSA as a likely upstream mediator of *p21* induction in KO‐MEFs under the l‐Ser‐depleted condition. Although doxSA was first identified as an anti‐cancer compound from the marine clam *Spisula polynyma* and has anti‐proliferative and apoptosis‐inducing activities 17, 18, 19, the molecular mechanisms underlying cell growth inhibition remain largely unknown. Unlike in marine clams, doxSA and other deoxysphingolipids are rarely present in mammalian plasma or tissues but were found recently in the plasma of patients with hereditary sensory and autonomic neuropathy type I (HSAN1) 16. HSAN1 is caused by missense mutations in *SPTLC1*, the subunit of SPT that catalyzes condensation of palmitoyl‐CoA with l‐Ser, the first step in sphingolipid synthesis 16. HSAN1‐associated mutations in *SPTLC1* cause a shift in the substrate specificity of SPT, which allows condensation of palmitoyl‐CoA with l‐Ala or Gly instead of l‐Ser 16, 25. We have just recently found that l‐Ser deficiency caused by loss of *Phgdh* in MEFs together with external l‐Ser limitation leads to the accumulation of doxSA, which showed a 7.1‐fold and a 30.6‐fold increase in KO‐MEFs after a 6‐h and 24‐h incubation, respectively, under the l‐Ser‐depleted culture condition as compared with those under the l‐Ser‐supplemented condition, presumably through alterations in SPT activity that are due to the relative increase in l‐Ala content over l‐Ser 8. Therefore, our data provide evidence that *de novo* l‐Ser synthesis functions as a metabolic gatekeeper to prevent an influx of l‐Ala into sphingolipid synthesis by supplying enough l‐Ser to SPT for the subsequent production of doxSA leading to activation of p38 MAPK–p21 in normal cells when the external l‐Ser supply is limited. Given that *p21* induction was not completely repressed by the treatment with the SPT inhibitor d‐Ser, it should be pointed out that doxSA appears not to be the sole regulator directing the elevation in *p21* mRNA in response to l‐Ser depletion. A recent study demonstrated that l‐Ser availability is sensed by the GCN2–eIF2α–ATF4 pathway 26. Consistent with this finding, we have observed that this pathway is activated in KO‐MEFs under the l‐Ser‐depleted condition 6, and there is a report showing that p21 expression is GCN2‐dependent in GCN2−/− MEFs under l‐Leu limitation 27. Therefore, we cannot exclude the possibility that the GCN2–eIF2α–ATF4 pathway might also participate in *p21* induction in l‐Ser‐depleted KO‐MEFs.

Independent of activation of p38 MAPK via doxSA that is one of adaptive response to l‐Ser deficiency for cell survival, intracellular l‐Ser was also required to preserve Ccnd1 levels and Rb phosphorylation, which are prerequisites for progression through the G1/S transition 21, 22. As mentioned above, consistent with a recent study 26, we have observed that translation initiation is inhibited in KO‐MEFs in the early stages of l‐Ser deficiency by increased phosphorylation (i.e., activation) of eIF2α, and in the later stages by induced expression of 4E‐BP1 6. Given that Ccnd1 has a rapid turnover rate, with an approximate half‐life of 24 min under normal proliferating conditions 28, prolonged inhibition of translation initiation could account for the reduction in Ccnd1 and the simultaneous decrease in phosphorylated Rb in l‐Ser‐depleted KO‐MEFs. Reduced Ccnd1 levels are found in the human glioma cell lines U87 and U251 when *PHGDH* expression is suppressed by shRNA 29. Stress‐induced accumulation of ceramide promotes dephosphorylation of Rb by activating the serine/threonine protein phosphatase PP1 30. Thus, it seems likely that the phosphorylation of Rb is regulated by intracellular levels of l‐Ser and its metabolite ceramide through distinct mechanisms.

l‐Ser deficiency disorders (SDD) are human inborn‐errors of *de novo* l‐Ser synthesis, which caused by mutations in *PHGDH* [MIM 601815], *PSAT1* [MIM 610992], and *PSPH* [MIM 614023] 31, 32. These patients exhibit common clinical features of neurodevelopmental symptoms such as congenital microcephaly, mental retardation, hypomyelinationa, and intractable seizures. Recent genetic study also identified two missense mutations in *PHGDH* that are associated with Neu‐Laxova syndrome 1 (NLS1 [MIM 256520]) 33, an autosomal‐recessive genetic disorder characterized by intrauterine growth retardation, severe malformation of multiple organs particularly the central nervous system and skin, and perinatal or early postnatal death. Subsequent genetic studies of NLS further confirmed stop‐codon mutation in *PHGDH* and missense mutations in *PSAT1* (NLS2 [MIM 616038]) and *PSPH* genes in other NLS patients 34. Shaheen and coworkers pointed out that some clinical symptoms of NLS are remarkably similar to various growth retarded phenotypes including brain malformation and lethality of conventional *Phgdh* KO mouse embryos 5. Our recent study demonstrated that KO‐MEFs used in this study accumulated several 1‐deoxysphingolipids including doxSA under l‐Ser deprived condition, and doxSA exerted detrimental effects on cell proliferation and survival of MEFs and other cells at micromolar concentration 8, leading the possibility that doxSA and other 1‐deoxysphingolipids may also participate in severe tissue malformations seen in NLS patients. Although clinical symptoms of SDD and NSL differ substantially, KO‐MEFs and conventional *Phgdh* KO mouse embryos can serve as a useful *in vitro* and *in vivo* models, respectively, to understand the molecular pathogenesis of NLS and also approach to effective strategy for preventing and treating their cell/tissue abnormalities.

There is growing interest in manipulating l‐Ser synthesis as a therapeutic intervention for cancer treatment. This interest is based on recent findings that *PHGDH* expression is enhanced in some cancer cells because of amplification of chromosome 1, which provides metabolic advantages that contribute to tumorigenesis 2, 3, 4. Interestingly, cancer cells with an amplified *PHGDH* genomic region or high levels of PHGDH appear to principally use the *de novo* l‐Ser synthetic pathway to boost intracellular production of α‐ketoglutarate from glutamate/glutamine 2. Despite the potential of l‐Ser deficiency to repress proliferation of cancer cells *in vitro* and *in vivo*, our data suggest that l‐Ser deficiency produced by manipulating the *de novo* l‐Ser synthetic pathway together with a limited external l‐Ser supply can also cause cell cycle arrest and a strong stress response in normal rapidly proliferating cells. Hence, further studies are needed to better define the molecular mechanisms underlying the effect of l‐Ser deficiency on cellular signaling programs and its toxicity to normal and cancer cells.

## Author contributions

TS, YH and SF designed the study and wrote the paper. TS, YK, WK, YA, KE, MH, MU and YK performed the cell culture experiments. TO contributed Phgdh‐transduced KO‐MEFs. WK, TS and HK performed microarray experiments. All authors analyzed the results and approved the final version of the manuscript.

## Supporting information


**Fig. S1.** l‐Ser deficiency diminishes Ccnd1 and phosphorylated Rb, whereas p38 MAPK and doxSA are not involved in the expression of Ccnd1 and Rb.
**Fig. S2.** Schematic representation of intracellular cascades activated in response to L‐Ser deficiency.
