# Supplementary material for: Adaptive response to l‐serine deficiency is mediated by p38 MAPK activation via 1‐deoxysphinganine in normal fibroblasts
Source: FEBS Open Bio. 2016 Mar 3;6(4):303–16. doi: 10.1002/2211-5463.12038 (PMC4821351; doi:10.1002/2211-5463.12038)
Supplement: Supplementary file 1 — Fig. S1. l‐Ser deficiency diminishes Ccnd1 and phosphorylated Rb, whereas p38 MAPK and doxSA are not involved in the expression of Ccnd1 and Rb. Fig. S2. Schematic representation of intracellular cascades activated in response to L‐Ser deficiency. [file FEB4-6-303-s001.pdf]

Supplementary information

Supplementary Fig. 1:

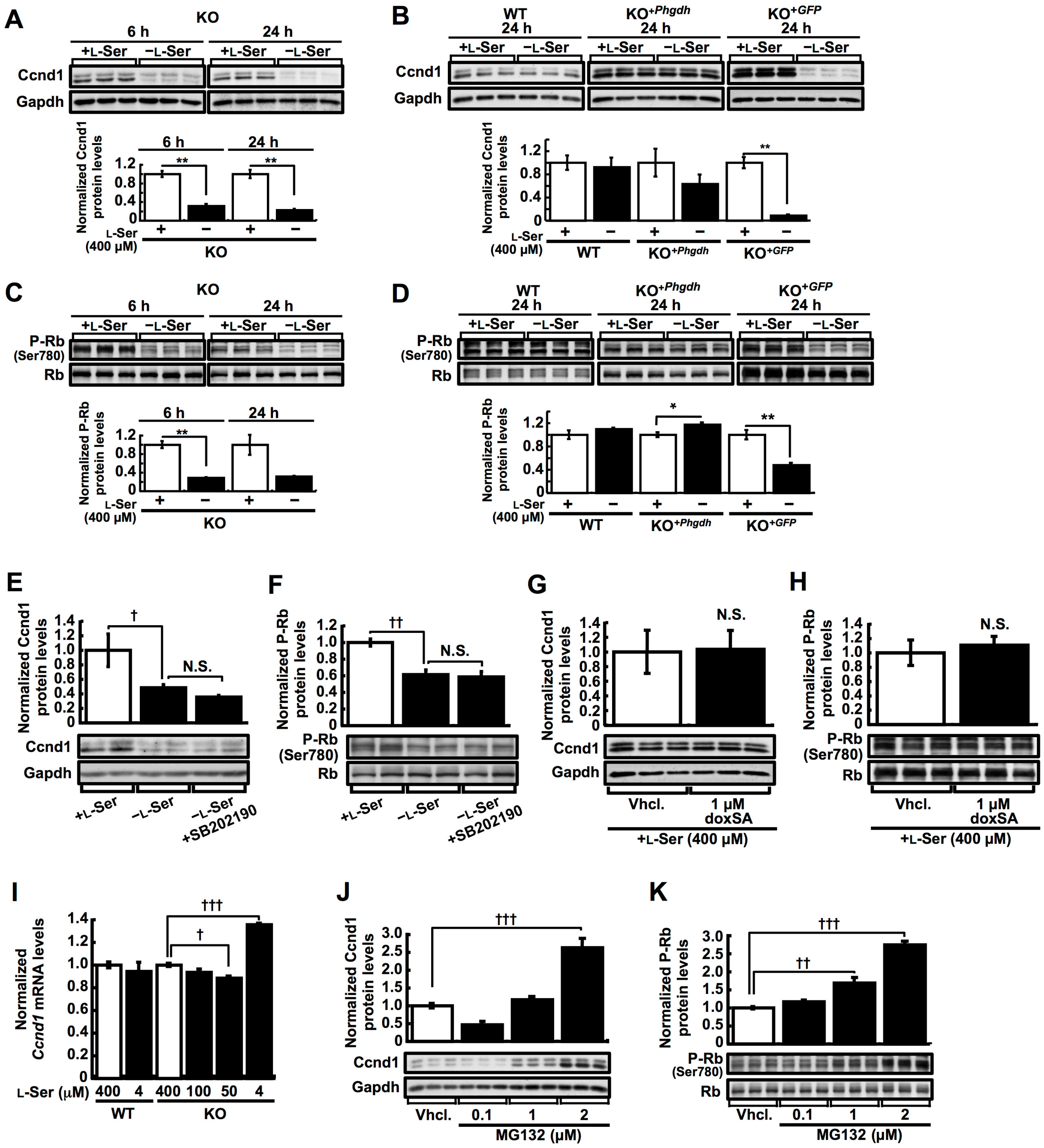

Supplementary Fig. 1: L-Ser deficiency diminishes Ccnd1 and phosphorylated Rb, whereas p38 MAPK and doxSA are not involved in the expression of Ccnd1 and Rb.

(A–D) Ccnd1 (A and B) and the phosphorylation (P) levels at Ser 780 of Rb and total Rb levels (C and D) were examined in KO-MEFs cultured for 6 or 24 h under the L-Ser–depleted or –supplemented condition, and in WT-MEF, KO-MEF^{+Phgdh}, and KO-MEF^{+GFP} cells cultured for 24 h under the L-Ser–depleted or –supplemented condition. Student's *t*-test, $*p < 0.05$, $**p < 0.005$. (E and F) KO-MEFs were cultured for 6 h under the L-Ser–supplemented or –depleted condition with or without the p38 MAPK inhibitor SB202190, and Ccnd1 (E) and phosphorylated Rb (F) levels were then analyzed. Dunnett's post-hoc test, $^{\dagger}p < 0.05$, $^{\dagger\dagger}p < 0.005$; $n = 4$ each; N.S., not significant. (G and H) KO-MEFs were cultured for 24 h with 1 μM doxSA under the L-Ser–supplemented condition, and Ccnd1 (G) and phosphorylated Rb (H) levels were then evaluated. (I) WT- and KO-MEFs were cultured for 6 h under the L-Ser–depleted or –supplemented condition, and *Ccnd1* mRNA was measured. Dunnett's post-hoc test, $^{\dagger}p < 0.05$, $^{\dagger\dagger\dagger}p < 0.0005$. (J and K) Ccnd1 and phosphorylated Rb levels were examined in KO-MEFs cultured for 6 h under the L-Ser–depleted condition in the presence or absence of the proteasome inhibitor MG132. Dunnett's post-hoc test, $^{\dagger\dagger}p < 0.005$, $^{\dagger\dagger\dagger}p < 0.0005$. Representative western blots show two or three samples for each treatment.

Supplementary Fig. 2:

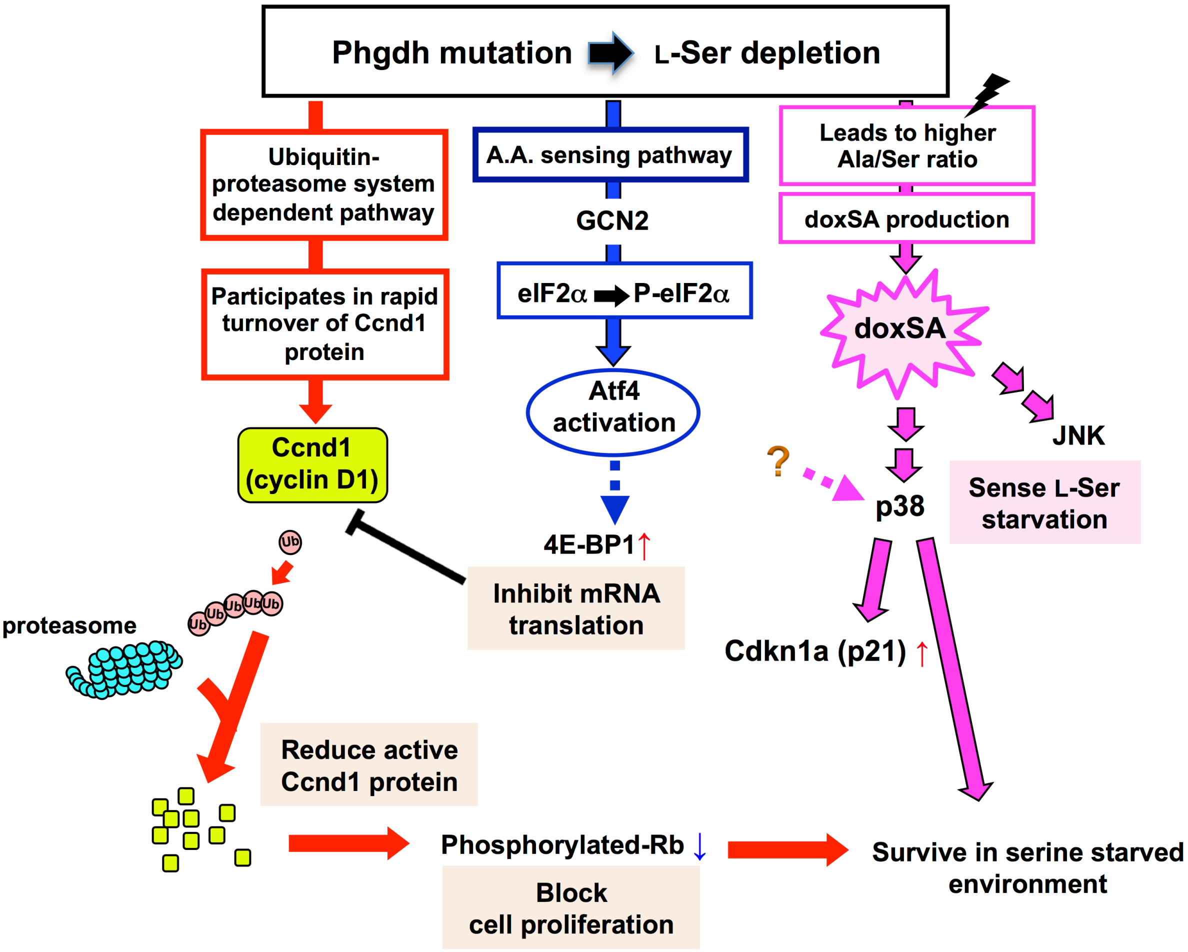

Supplementary Fig. 2: Schematic representation of intracellular cascades activated in response to L-Ser deficiency.

Reduced availability of L-Ser leads to activation of multiple cascades in order to adapt to and survive in L-Ser starved environment. The GCN2-eIF2α-ATF4 pathway (amino acid (A.A.) sensing pathway), leading to reduction of protein synthesis and negative regulation of cell cycle modulators, thereby resulting in cell cycle arrest. p38 MAPK activation through doxSA production may participate in cell survival under the L-Ser deficient condition.
